# Spermidine Supplementation Effectively Improves the Quality of Mouse Oocytes After Vitrification Freezing

**DOI:** 10.3390/antiox14020224

**Published:** 2025-02-16

**Authors:** Li Wang, Weijian Li, Yalan Liu, Airixiati Dilixiati, Zhanzhan Chang, Yibai Liang, Yuhan Wang, Xiuling Ma, Ling Tang, Zhi He, Yuan Zhang, Xuguang Wang

**Affiliations:** Xinjiang Key Laboratory of Equine Breeding and Exercise Physiology, College of Animal Science, Xinjiang Agricultural University, Urumqi 830052, China

**Keywords:** vitrification freezing, mitochondria, oxidative stress, single-cell transcriptome, oocytes, spermidine

## Abstract

The cryopreservation of oocytes through vitrification is imperative for the conservation of livestock germplasm resources. However, as oocytes exhibit significant oxidative stress and organelle damage following vitrification freezing, it is crucial to optimise the vitrification conditions to mitigate the deleterious effects of freezing. In this study, we demonstrated that spermidine has been showed to enhance oocyte survival after vitrification freezing (92% ± 4% vs. 82% ± 3%, *p* < 0.01) and blastocyst formation after freezing for in vitro fertilisation (14.86% ± 7% vs. 6% ± 3, *p* < 0.05). Spermidine supplementation rescued 47.3% of dysregulated pathways, including ovarian steroidogenesis, and restored normal expression levels in 43.3% of aberrantly expressed genes. Subsequent studies elucidated that spermidine effectively rescued mitochondrial dysfunction after vitrification, alleviated oxidative stress damage, and regulated intracellular calcium homeostasis. Consequently, we concluded that the addition of spermidine during vitrification freezing is an effective method to protect oocytes from freezing damage.

## 1. Introduction

Oocyte vitrification is a cryopreservation technique that employs rapid dehydration and ultra-rapid cooling in liquid nitrogen, utilising high concentrations of cryoprotective agents to achieve a glass-like state. As a significant technology in the field of embryo bioengineering, vitrification freezing has been demonstrated to be an effective method of preserving the germplasm resources of outstanding female animals [1]. Furthermore, this method enables efficient long-distance transport and subsequent utilisation of oocytes, overcoming geographical limitations in reproductive applications. Notably, this approach significantly reduces both breeding cycles and associated operational costs, thereby offering a practical solution for enhancing reproductive efficiency in clinical and agricultural settings [2]. The vitrification freezing method is distinguished from slow freezing by the rapid formation of a vitrification-like state around the oocytes without the formation of ice crystals and therefore has a higher survival rate [3]. However, oocytes are distinguished by their considerable size, substantial water content, singular cell state, and distinctive chromosomal configuration, which render them more challenging to freeze [4].The primary issue encountered by oocytes following vitrification freezing is the heightened level of reactive oxygen species within the oocytoplasm. This has been shown to result in impaired intracellular organelle function, including the spindle, the endoplasmic reticulum, and the mitochondria, which can consequently lead to a reduction in fertilisation and developmental capacity of the frozen oocytes [5]. It has been demonstrated that during the process of vitrification freezing, characterised by sudden temperature fluctuations, the antioxidant defence system of the oocyte is significantly compromised, with the mitochondria, as the primary line of antioxidant defence, being the initial target of reactive oxygen species [6]. This results in the impairment of their structural and functional integrity [7]. Consequently, the incorporation of antioxidants into the oocyte culture system and the vitrification-freezing process has been demonstrated to enhance the level of oxidative stress and developmental capacity of oocytes following freezing [8]. Earlier research has established that resveratrol [9], glutathione [10], and melatonin [11] can function as antioxidants during the cryopreservation of mammalian oocytes, thereby enhancing the antioxidant defence system of the oocytes. Nevertheless, the efficacy of these pharmaceuticals is restricted to the enhancement of antioxidant levels following the freezing process. Consequently, there is a necessity to identify a novel protective agent that can not only elevate the antioxidant level but also influence the morphology distribution of organelles and gene expression.

Spermidine (Spd) is an intermediate of polyamine compound that has been found in most tissues and cells, including semen and ovaries [12]. It has been demonstrated to effectively remove intracellular free radicals as an endogenous potent antioxidant [13] and also participates in a wide range of cellular biological processes by enhancing mitochondrial autophagy [14]. It has been reported that yeast, fruit flies, and nematodes can induce cellular autophagy and prolong lifespan by exogenous supplementation of spermidine [15,16]. Recent studies have found that spermine supplementation of sows during gestation increases the number of healthy piglets and promotes the development of placental tissues [17]. Conversely, the development of diabetes mellitus and its complications can be inhibited by exogenous spermidine and *RIPK1* inhibitors during the ageing process [18]. Furthermore, spermidine has been identified as a pivotal substance in ovarian anti-ageing and antioxidant processes, with the use of spermidine having the potential to enhance the quality of oocytes in aged mice by promoting mitochondrial autophagy [19]. Despite the aforementioned advantages of spermidine, there are few studies on its use as an antioxidant in vitrification and cryopreservation of oocytes.

In this study, it was demonstrated that the incorporation of spermidine during the cryopreservation of maturated MII oocytes in vitro led to an enhancement in the survival rate of oocytes post-vitrification and the development rate of in vitro fertilisation. Transcriptome sequencing revealed that supplementation of spermidine led to the restoration of 47.3% of the aberrant pathways, including ovarian steroidogenesis, and 43.3% of the abnormally expressed genes. Furthermore, the addition of spermidine during the vitrification process led to a reduction in oxidative stress, thereby mitigating damage to organelles such as mitochondria and the endoplasmic reticulum.

## 2. Materials and Methods

### 2.1. Animals and Materials

This study was approved by the Animal Ethics Committee of Xinjiang Agricultural University (2024026, 4 February 2024).

The test animals utilised in this experiment were Kunming white strain mice (purchased from Xinjiang Medical University Animal Experiment Centre), which were maintained in separate cages for males and females with a standard laboratory chow and unrestricted water intake, a light period of 12 h, and a growing environment of 18–25 °C. The mice were used for the experiment after one week of acclimatising to the environment of the mouse house.

Unless stated otherwise, all reagents were procured from Sigma (St. Louis, MO, USA), and Petri dishes and associated consumables were obtained from Coning Inc. (Corning, NY, USA).

### 2.2. Oocyte Collection and Maturation

The mice were prepared for supernumerary ovulation following the implementation of certain modifications to the previously described methods [20]. Mice with healthy bodies and glossy hair, aged 5–8 weeks, were selected for superovulation treatment. They were injected with pregnant mare serum gonadotropin (PMSG), which is used to stimulate superovulation in mice by promoting follicular development. The dose was 10 IU, given at 18:00. After 46–48 h, the ovaries on both sides of the uterus were collected into well-balanced operative droplets (PBS + 0.3% BSA). The fat from the ovaries were removed under the stereomicroscope, and the follicles on the ovaries were subsequently scratched by forceps and syringe. This process resulted in the release of the oocytes from the follicles. The oocytes were then meticulously selected based on their morphology, shape, and the presence of germ-vesicle. Following this, the oocytes were washed and transferred to mature Petri dishes (α-MEM + 10% FBS + 10 ng/mL EGF) that had been equilibrated in a CO_2_ incubator for a minimum of 6 h. Each microdrop, containing 60 μL of maturation medium, was loaded with thirty oocytes. The dish including the oocytes was then placed into an incubator set to a temperature of 37 °C and a CO_2_ concentration of 5%, where the oocytes were cultivated for a period of 12 h.

### 2.3. Vitrification Freezing and Warming of Oocytes

The Open Pulled Straw (OPS) should be prepared at least one day in advance. One hour prior to the commencement of the experiment, the operating solution (DPBS + 0.3% BSA), equilibrium solution (DPBS + 10% EG + 10% DMSO), and freezing solution (DPBS + 20% EG + 20%DMSO + 0.5 mol/L Sucrose + 0.1 mol/L Ficoll) must be prepared. Spermidine (Item No S0266, Sigma) was added to the equilibrium solution and freezing solution at 0, 25, 50, and 100 μmol/L and mixed thoroughly for the experimentation [19]. Following a 12 h in vitro maturation period, matured oocytes that exhibited a first polar body and a uniform cytoplasm were collected for vitrification freezing. The oocytes were equilibrated in the equilibration solution for approximately 35 s and then immediately transferred to the vitrification-freezing solution for 20 s. Subsequently, the oocytes were transferred directly to the OPS and stored in liquid nitrogen for freezing. The oocytes were stored in liquid nitrogen for a minimum of one week prior to warming and subsequent experimentation.

**Figure 1:**
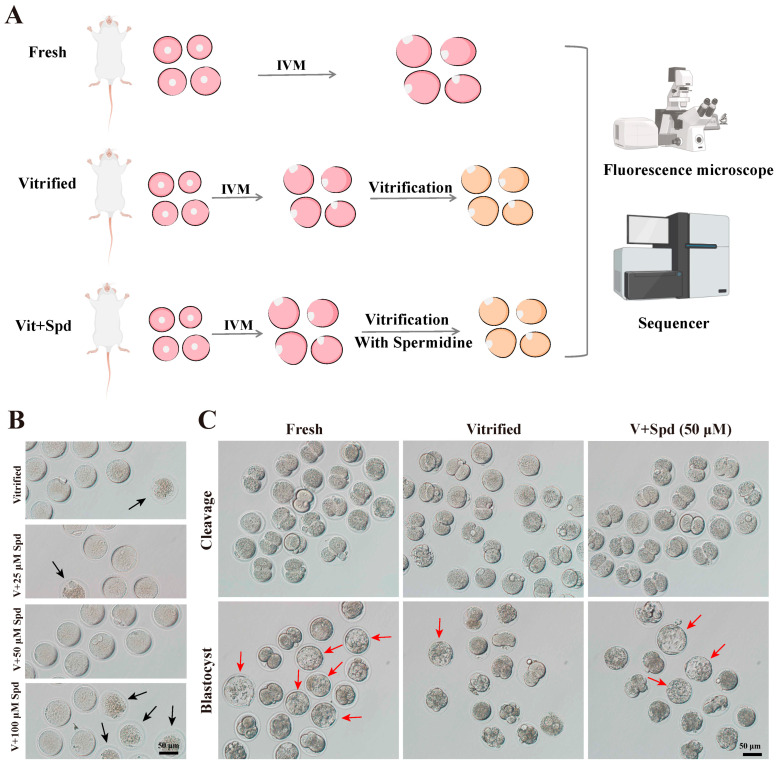
Effects of spermidine supplementation on survival rate and embryonic development after freezing: (**A**) Test flow chart. (**B**) Effect of different concentrations of spermidine on matured oocytes after vitrification freezing. Black arrows indicate dead oocytes. (**C**) Effects of fresh group, vitrification-frozen group, and addition of 50 μmol/L spermidine on in vitro fertilisation development rate. Red arrows indicate dead oocytes blastocyst.

The warming solution (DPBS + 0.5 mol/L Sucrose + 3% BSA) and recovery solution (DPBS + 0.5% BSA) were prepared in advance for warming. Thereafter, the OPS tubes containing the oocytes were retrieved from liquid nitrogen, the oocytes were expelled, and they were then incubated in the warming solution for a period of five minutes. Thereafter, they were transferred to the recovery solution for a washing process. Following a minimum of three washes, the oocytes were transferred to equilibrated recovery dishes for a 2 h period to facilitate subsequent experimentation. All operations were performed on a 37 °C hot plate, and all solutions were kept at 37 °C. Following a recovery period of two hours, the morphology of the oocytes was observed under a stereomicroscope. Oocytes exhibiting uniform, intact, and refractive cytoplasm were marked as surviving oocytes, while oocytes manifesting overall shrinkage of cytoplasm and reduction in volume were categorised as dead oocytes (Figure 1B). Survival rate = (number of surviving oocytes/total oocytes) × 100%.

### 2.4. In Vitro Fertilisation and In Vitro Culture

In the week preceding the warming of the oocytes, the males and females (in a 1:2 ratio) were placed in a common case. Following a second day of checks for vaginal embolism, male mice were housed separately for in vitro fertilisation experiments. Semen was collected from the epididymis of both sides of the males 1 h before warming and placed in a pre-prepared sperm capacitation dish. The resuscitated oocytes were then taken out and washed at least three times in a dish of fertiliser solution before waiting for fertilisation. Following the completion of sperm capacitation, the spermatozoa were introduced to the fertilised droplets containing the oocytes and then left to incubate for a period of 3–6 h. Subsequent to the completion of the incubation period, all spermatozoa were washed away and observed for the presence of prokaryotic nuclei formation. The washed oocytes were then transferred to pre-made in vitro dishes and cultivated in a CO_2_ incubator. The culture conditions were set to 37 °C, saturated humidity, 5% CO_2_, and the rate of cleavage was observed for 24 h and the rate of blastocysts for 96 h.

### 2.5. Detection of Intracellular Reactive Oxygen Species and Glutathione Levels

Matured oocytes from each group were washed thrice in the operating solution, after which reactive oxygen species (ROS) and glutathione (GSH) were quantified using 15 μmol/L 2′,7′-DCFH-DA and 15 μmol/L Cell Tracker Blue probe (Invitrogen, Carlsbad, CA, USA). Following a 20 min incubation period, the oocytes were washed with the operating solution and then placed on slides for observation and microscopic imaging using an inverted fluorescence microscope. Subsequent to this, the oocytes were analysed for GSH and ROS using ImageJ software (version 1.54f), and the average fluorescence intensity was calculated.

### 2.6. Mitochondrial Membrane Potential Assay

Matured oocytes from each group were then subjected to an incubation in a 10 mmol/L JC-1 working solution (Solarbio, Beijing, China) at a temperature of 38.5 °C and a 5% CO_2_ for a period of 20 min. Following this, the oocytes were washed with the operating solution. Thereafter, the oocytes were placed on slides in the inverted fluorescence microscope for observation and microscopic imaging. The mitochondrial membrane potential of the oocytes was analysed by ImageJ software (version 1.54f), and the average fluorescence intensity was calculated. The value of mitochondrial membrane potential was calculated as the ratio of red fluorescence (aggregates) to green fluorescence (monomers).

### 2.7. Assessment of Mitochondrial Function and ATP Levels

Matured oocytes from each period were incubated in 5 μmol/L MitoTracker GreenFM (Invitrogen, USA) and MitoSOX Red (Invitrogen, USA) staining solution at 38.5 °C for 20 min in 5% CO_2_. At the end of the incubation period, the oocytes were washed with the operating solution. The mitochondria were enumerated, and the even distribution of mitochondria in the cytoplasm of the oocytes was considered normal, while the mitochondria in clusters or unevenly distributed in the cytoplasm of the oocytes were considered abnormal. ATP levels were quantified by using a BODIPY™ FL ATP probe (Invitrogen, USA) at 38.5 °C for 20 min in 5% CO_2_. Following this, the oocytes were placed on slides and observed and photographed under the inverted fluorescence microscope.

### 2.8. Detection of Endoplasmic Reticulum Distribution

Matured oocytes from different groups were incubated in 5 μmol/L ER-Tracker Red (Invitrogen, USA) at 38.5 °C with 5% CO_2_ for 30 min. The oocytes were then washed with the operating solution at the end of the incubation, after which they were placed on slides for observation and photographed with the inverted fluorescence microscope. The even distribution of endoplasmic reticulum in the cytoplasm of oocytes was considered normal, while the endoplasmic reticulum in clusters or unevenly distributed in the cytoplasm of oocytes was considered abnormal.

### 2.9. Oocyte Calcium Assay

Matured oocytes from the various groups were subjected to an incubation process, during which they were exposed to 5 μmol/L Rhod-2 AM (Invitrogen, USA) and Mag Fluo-4 AM (Invitrogen, USA) in a 5% CO_2_ atmosphere at a temperature of 38.5 °C for a duration of 15 min. Following the completion of the incubation period, the oocytes were thoroughly washed with the operating solution. Subsequent to the washing step, the oocytes were placed on slides and observed and photographed under an inverted fluorescence microscope. The intra-oocyte calcium and mitochondrial calcium content of the oocytes were then calculated by utilising ImageJ software (version 1.54f).

### 2.10. Immunofluorescence Staining

Matured oocytes from each group were fixed in 4% paraformaldehyde (PFA) for 30 min at room temperature. Subsequently, they were permeabilized with 0.5% Triton X-100 in PBS (Solarbio, Beijing, China) for 1 h at room temperature, followed by blocking with 3% BSA solution for 1 h at room temperature. At the end of the blocking process, the oocytes were incubated with anti-α-tubulin-FITC (Sigma, USA) for 1 h, and then washed three times with 0.1% Tween 20-PBS. Finally, the oocytes were re-stained with DAPI (Sigma, USA) for 6 min, after which they were washed with the operating solution and placed on slides for observation and microscopic imaging under an inverted fluorescence microscope. The presence or absence of specific morphological abnormalities of the spindle was then determined. Abnormal spindles were defined as multipolar, abnormally enlarged, and abnormally arranged microtubules, while normal spindles were defined as bipolar, broad in the middle and narrow at both poles, and normally arranged microtubules.

### 2.11. RNA Sequencing Analysis

Fresh, control vitrified-frozen, and spermidine-treated vitrified-frozen oocytes were collected in three replicates per group. Following lysis and amplification, the construction of cDNA libraries was initiated. The Illumina NovaSeq 6000 platform (San Diego, CA, USA) was then utilised for sequencing, employing a PE 150 configuration. Upstream analysis was conducted using the Linux CentOS 7 operating system. The obtained raw data were subjected to detailed removal of sequencing adapters and bases of low quality by employing the fastp software (version 0.23.4). Subsequently, the reference genome index was constructed by utilising Hisat2 and then subjected to mapping with the mouse reference genome (GCF_000001635.27_GRCm39). Following this, the gene level counts were calculated using the Rsubread. The Variance Stabilising Transformation (VST) approach, leveraging the negative binomial distribution and implemented through the DESeq2 package, was employed for counts normalisation and subsequent statistical analysis of differentially expressed genes (DEGs). Image plotting was performed using the ggplot2 package in R (version 4.4.2). GO, KEGG, and GSEA enrichment analyses were performed using the clusterProfiler package in R.

### 2.12. Statistical Analyses

The data are expressed as the mean ± standard deviation, with a minimum of three independent replicates per experiment and a minimum of three mice per replicate. Statistical analyses were performed using a one-way ANOVA and chi-square test. Significance test analyses were carried out using the built-in pipeline of prism (version 9.5.1). Multiple tests were performed using Tukey’s method. ns indicates *p* > 0.05, * indicates *p* < 0.05, and ** indicates *p* < 0.01.*** indicates *p* < 0.0001. Correlation analyses were performed using a Pearson correlation coefficient calculation method. Hierarchical clustering was performed using ward.D2 algorithm.

## 3. Results

### 3.1. Spermidine Improves Oocyte Survival and Development After Vitrification Freezing

The experiment commenced with an investigation into the impact of spermidine incorporation into equilibration and freezing solutions on the survival rate of mouse oocytes following vitrification freezing (Figure 1A). Spermidine was added at 0, 25, 50, and 100 μmol/L during equilibration and freezing(Figure 1B). As demonstrated in Table 1, the incorporation of 50 μmol/L spermidine resulted in a significant enhancement in the survival rate of post-freezing oocytes in comparison to the control (*p* < 0.01). However, when the concentration was elevated to 100 μmol/L, the survival rate exhibited a substantial decline compared to the control (*p* < 0.01). Subsequently, the effect of 50 μmol/L spermidine on in vitro fertilisation following vitrification was investigated (Figure 1C). As demonstrated in Table 2, the cleavage and blastocyst rates of in vitro-fertilised oocytes following vitrification freezing were significantly lower than those of the unfrozen group (Fresh Control) (*p* < 0.01). In contrast, the addition of 50 μmol/L spermidine to both the equilibrium and freezing solutions had no significant impact on the cleavage rate (*p* > 0.05), yet it resulted in a significant increase in the blastocyst rate (*p* < 0.05).

### 3.2. Effect of Spermidine Addition on Transcriptional Profiles

**Figure 2:**
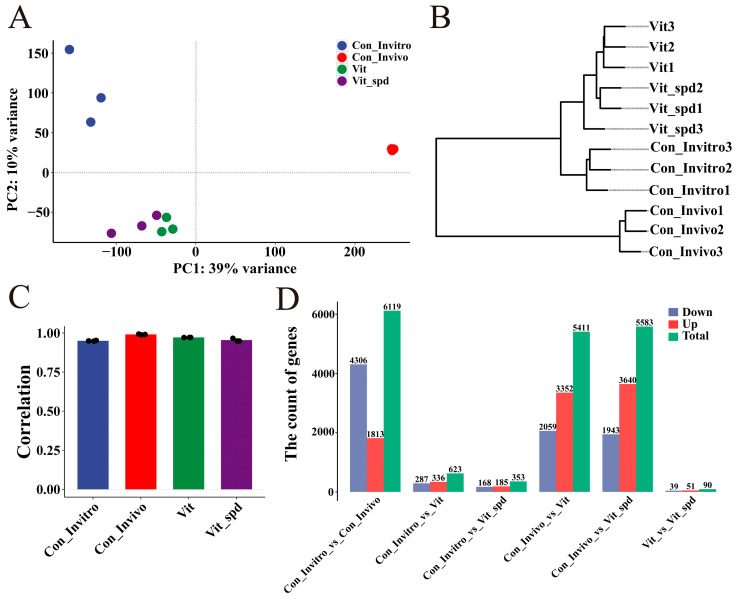
Differentially expressed genes before and after vitrification freezing: (**A**) PCA plot showing the distribution of Con_invitro, Con_vivo, vit, and vit + Spd groups after dimensionality reduction. (**B**) Hierarchical clustering of individual samples from the Con_invitro, Con_vivo, vit, and vit+Spd groups using the ward.D2 method, observing distances between and within groups. (**C**) The correlation bar graph shows the correlation of the groups. (**D**) Differently expressed gene analysis using Deseq2 was followed by bar graphs showing the differentially expressed genes between the Con_invitro, Con_vivo, vit, and vit+Spd groups.

We investigated the mechanism of the efficiency enhancement of spermidine on vitrification-frozen mouse oocytes. We performed transcriptome analyses on matured oocytes from in vitro matured (Con_Invitro), in vivo matured (Con_Invivo), vitrification-frozen (Vit), and vitrification-frozen groups with the addition of spermidine (Vit + Spd), respectively. A correlation analysis showed that the correlation of each group was greater than 95% (Figure 2C). A principal component analysis showed clustering between repeats within the Con_Invitro, Con_Invivo, Vit, and Vit +Spd groups and dispersion between groups (Figure 2A). The same result was found for hierarchical clustering (Figure 2B). When all points are mapped to PC1, it is clear that Con_Invitro and Con_Invivo are distributed on both sides, while Vit and Vit + Spd are in the middle, and Vit + Spd is closer to Con_Invitro compared to Vit. Projection of all data points onto PC2 reveals three distinct clusters: Con_Invitro, Con_Invivo, and the vitrified-frozen cluster (comprising Vit and Vit + Spd). Notably, the Con_Invivo cluster occupies a central position, flanked by the Con_Invitro and vitrified-frozen clusters on either side (Figure 2A).

**Figure 3:**
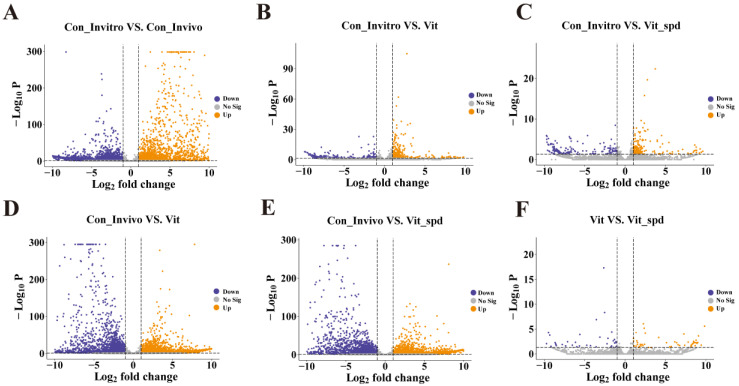
Volcano plots between Con_Invitro, Con_Invivo, Vit, and Vit +Spd groups.(**A**–**F**) The relationship between log_2_ (fold change) and −Log_2_ *p*-value between each of the two groups is shown using a volcano plot.

The differential expression of genes was then counted (Figure 2D). We screened 623 differentially expressed genes (DEGs) between Con_Invitro and Vit, of which 336 were up-regulated and 287 were down-regulated. Between Con_Invitro and Vit + Spd, the DEGs decreased to 353, of which 185 were up-regulated and 168 were down-regulated. And this result showed that treatment with Spd rescued 43.3% of the abnormal genes in Vit compared to Con_Invitro (Figure 3B,C). In contrast, the total number of DEGs between Con_Invivo and Vit was 5411, while the number of DEGs between Con_Invivo and Vit + Spd remained at 5583, showing no significant reduction in DEGs with treatment of the Spd. A total of 90 DEGs were found when comparing Vit with Vit + Spd (Figure 3).

### 3.3. Enrichment Analysis of Differentially Expressed Genes

**Figure 4:**
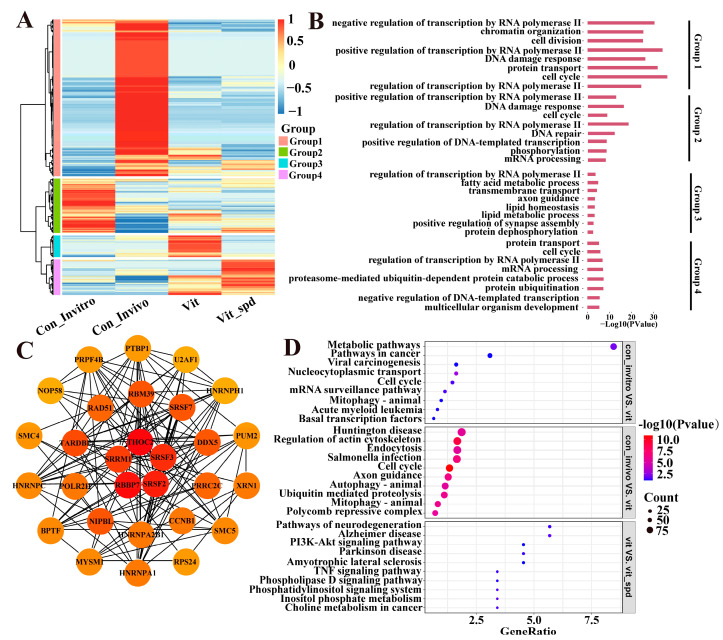
Heatmap, GO, and KEGG analysis: (**A**) The heatmap shows the overall distribution of all differentially expressed genes and clustered using gene expression into 4 clusters. (**B**) Functional annotation of genes within each cluster obtained from the A using the GO enrichment analysis database. (**C**) Differentially expressed genes were subjected to protein interaction network analysis and then screened for hub genes using the degree algorithm. (**D**) KEGG enrichment analysis of each group.

Subsequently, an investigation was conducted into all differentially expressed genes. Utilizing the heatmap, it was observed that all differentially expressed genes could be categorised into four distinct clusters (Figure 4A,B). Group 1 is a Con_Invivo-specific high-expression cluster, which is mainly related to chromatin organisation, regulation of transcription by RNA polymerase II, etc. Conversely, the expression levels of Group 2 genes were markedly diminished in the Vit compared to Con_Invitro. Notably, these genes remained refractory to downregulation, even in the presence of Spd. Functional analyses revealed that these genes are predominantly involved in critical cellular processes, such as the DNA damage response, mRNA processing, and DNA repair. Group 3 comprises a set of differential genes that were successfully rescued by the addition of Spd, and their main functions are regulation of transcription by RNA polymerase II, fatty acid polymerase II, and RNA polymerase II, as well as the processes of fatty acid metabolic, transmembrane transport, and protein dephosphorylation. The genes in Group 4 are characterised by their high level of expression and the inability to be rescued by the addition of Spd. These genes are associated with functions such as protein transport, proteasome-mediated ubiquitin-dependent protein catabolic processes, protein ubiquitination, and multifunctionality.

The KEGG enrichment analysis (Figure 4D) showed that differentially expressed genes in Con_Invitro versus Vit were mainly enriched in the pathways of metabolic pathways, pathways in cancer, viral carcinogenesis, nucleocytoplasmic transport, etc. Con_Invivo and Vit differentially expressed genes were mainly enriched in regulation of actin cytoskeleton, endocytosis, cell cycle, mitophagy–animal, and other pathways. In contrast, the differentially expressed genes of Vit and Vit_Spd were predominantly enriched in pathways associated with neurodegeneration, the Phosphatidylinositol 3-Kinase—Protein Kinase B signalling pathway(PI3K-Akt), the TNF signalling pathway, and the Phospholipase D signalling pathway. Finally, a Protein–Protein Interaction Network analysis (PPI) was performed on the differentially expressed genes (Figure 4C), and a total of five HUB genes were identified, namely THO complex subunit 2 (*THOC2*), Serine/Arginine-rich splicing factor 3 (*SRSF3*), Serine/Arginine-rich splicing factor 2 (*SRSF2*), Retinoblastoma binding protein 7 (*RBBP7*), and Serine/Arginine repetitive matrix 1 (*SRRM1*).

**Figure 5:**
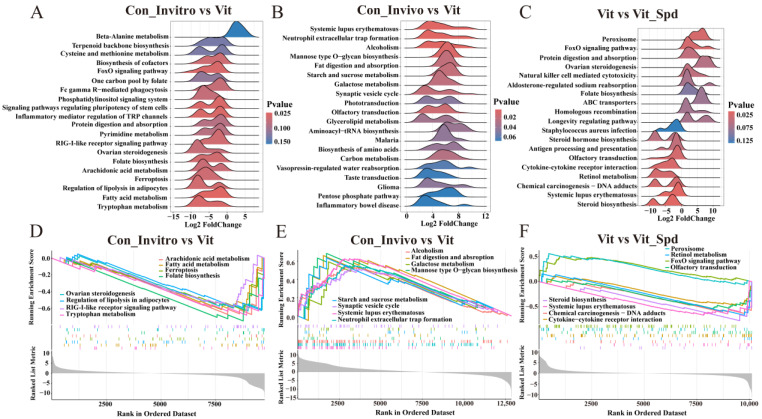
GSEA. (**A**–**C**) GSEA ridge plot. (**D**–**F**) GSEA enrichment score fold plot.

The following study was conducted with the objective of investigating the effect of freezing and Spd treatment on the pathways by all gene expressions. To this end, gene set enrichment analyses (GSEAs) were subsequently conducted, resulting in the generation of ridge plots and enrichment score line graphs, respectively. A comparison was made between the Con_invitro and Vit groups, and it was observed that, among the Top 20 ranked pathways, the majority exhibited a tendency towards down-regulation, with the exception of Beta-Alanine metabolism (Figure 5A,D). Upon observation, it was found that these pathways were predominantly enriched in the Con_Invitro and Vit groups, particularly in ovarian steroidogenesis, RIG-I-like receptor-signalling pathway, signalling pathways regulating pluripotency of stem cells, and a variety of metabolism-related pathways. In contrast, Con_Invivo exhibited a tendency towards up-regulation in all of the top 20 pathways in comparison to Vit (Figure 5B,E), predominantly concentrated in the pentose phosphate pathway, systemic lupus erythematosus, synaptic vesicle cycle, and a variety of amino acid and sugar metabolism processes. Vit exhibited up-regulation in 10 of the top 19 pathways and down-regulation in 9 compared to Vit_Spd, primarily enriched in Peroxisome, FoxO-signalling pathway, ovarian steroidogenesis, ABC transporters, homologous recombination, retinol metabolism, and other pathways. The results demonstrated that the addition of Spd during the freezing process was capable of rescuing approximately 47.3% of the aberrant pathways (Figure 5C), which is consistent with the results of Spd on the rescue of differentially expressed genes (Figure 3B,C). Based on the above results, it is clear that there is a large difference between in vivo matured and in vitro matured oocytes, and the vitrified frozen oocytes used in this experiment all originated from in vitro maturation. Therefore, all subsequent analyses were based on in vitro maturation as a control group.

### 3.4. Spermidine Reduces Oxidative Stress After Vitrification Freezing

**Figure 6:**
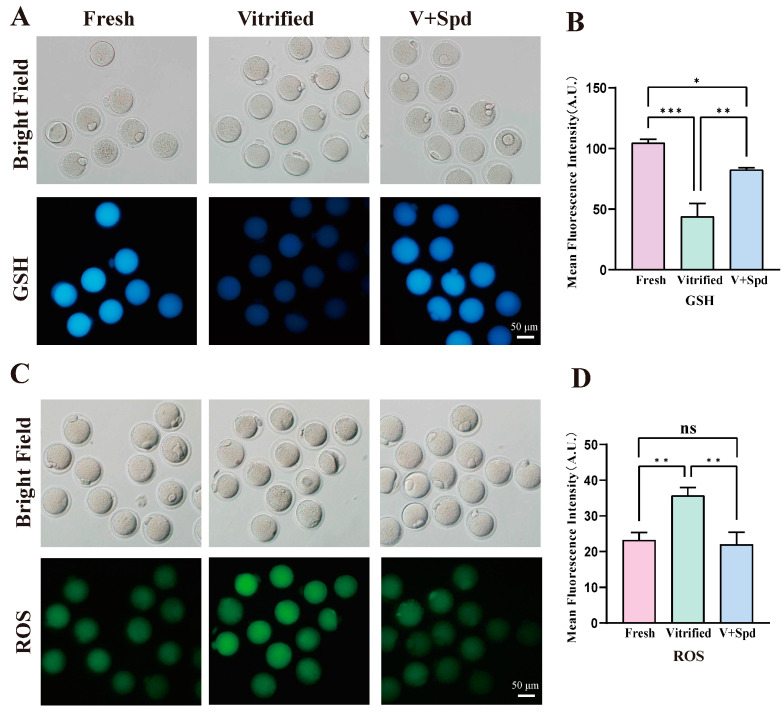
Effect of spermidine on oxidative stress in frozen oocytes: (**A**) The Cell Tracker Blue probe visualises GSH levels in oocytes, with higher fluorescence intensity indicating higher GSH levels. (**C**) ROS levels were visualised using DCFH-DA, with higher fluorescence intensity indicating higher ROS levels, and lower fluorescence intensity indicating lower ROS levels. (**B**,**D**) Histogram of fluorescence intensity statistics for each group, showed in Mean ± SD. ns indicates *p* > 0.05, * indicates *p* < 0.05, and ** indicates *p* < 0.01. *** indicates *p* < 0.0001.

Oxidative stress levels strongly and negatively correlated with oocyte recovery after vitrification freezing. Given the potent antioxidant properties exhibited by spermidine, we investigated its potential to mitigate the adverse effects associated with oocyte vitrification. We examined intracellular oxidative stress levels by assessing glutathione (GSH) and reactive oxygen species (ROS) levels. As Figure 6 clearly shows, GSH levels in vitrified oocytes were significantly lower than in the fresh group (*p* < 0.01), and ROS levels were significantly higher than in the fresh group (*p* < 0.01). This proved that vitrification freezing caused oxidative stress in oocytes. However, adding 50 μmol/L spermidine during the freezing process (V + Spd) led to a significant decrease in ROS levels (*p* < 0.01) and a substantial increase in GSH levels (*p* < 0.01). The results were clear that adding spermidine to the freezing process reduced ROS levels and increased GSH levels in vitrified-frozen oocytes.

### 3.5. Spermine Restores Mitochondrial Function in Vitrified Oocytes

**Figure 7:**
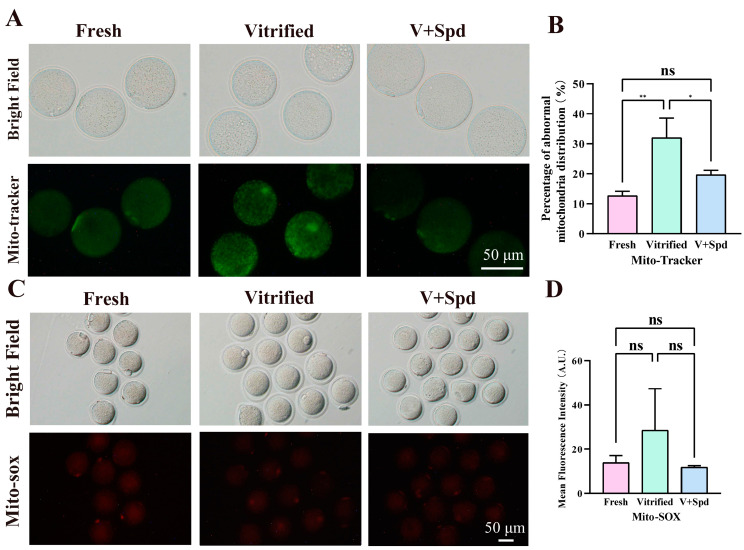
Effect of spermidine on mitochondrial distribution and reactive oxygen species levels in vitrified oocytes: (**A**) Mitochondrial distribution was visualised using Mito-tracker. Fluorescence evenly distributed in the cytoplasm was identified as a normal mitochondrial distribution as shown in the fresh group, while clusters of fluorescence showing irregularity were identified as an abnormal mitochondrial distribution as shown in the vitrified group. (**C**) Reactive oxygen species levels were visualised using Mito-sox, with higher fluorescence intensity indicating higher levels of reactive oxygen species. (**B**,**D**) Histogram of fluorescence intensity statistics for each group, showed in Mean ± SD. ns indicates *p* > 0.05, * indicates *p* < 0.05, and ** indicates *p* < 0.01.

**Figure 8:**
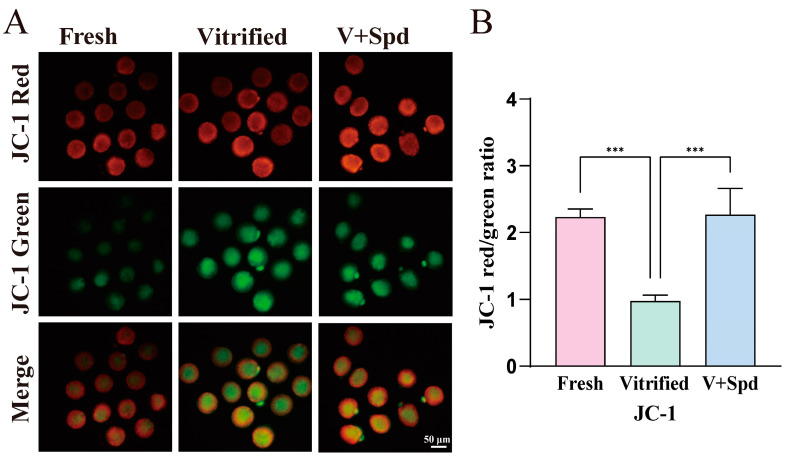
JC-1 was used to investigate the effect of vitrification freezing on mitochondrial membrane potential of oocytes. (**A**) Visualisation of mitochondrial membrane potential using the JC-1 probe. At high mitochondrial membrane potentials, JC-1 aggregates in the mitochondrial matrix, forming polymers that show red fluorescence (functional mitochondria). At low mitochondrial membrane potential, JC-1 fails to accumulate in the matrix of mitochondria, and then JC-1 is a monomer and shows green fluorescence (impaired mitochondria). (**B**) Histogram quantifying the ratio of red fluorescence (functional mitochondria) to green fluorescence (impaired mitochondria), showed in Mean ± SD. *** indicates *p* < 0.0001.

**Figure 9:**
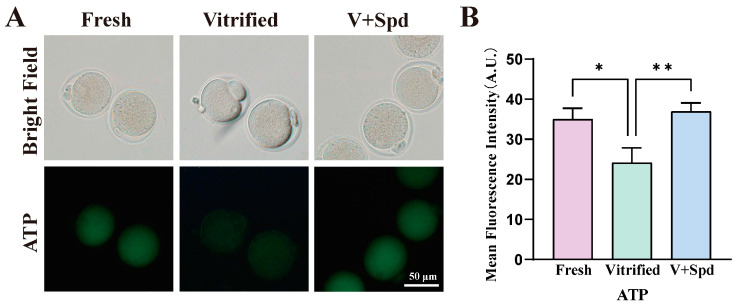
Effect of spermidine on the ATP content of vitrified oocytes: (**A**) Visualisation of ATP levels in oocytes using the BODIPY™ FL ATP Probe, with higher fluorescence intensity indicating higher ATP levels. (**B**) Histogram plots of fluorescence intensity for the fresh, vitrified and V+Spd groups, showed in Mean ± SD. * indicates *p* < 0.05, and ** indicates *p* < 0.01.

Mitochondria are the main organelles that produce reactive oxygen species and the main site of ATP production, which is essential for maintaining cellular homeostasis [21], and the decrease in ROS levels with the addition of spermidine may be one of the important factors in improving the survival of oocytes after vitrification freezing (Figure 6B,D). Therefore, we continued to explore the mitochondrial distribution, mitochondrial membrane potential, mitochondrial reactive oxygen species level, and ATP level. The results showed that the proportion of mitochondrial abnormality distribution in vitrified-frozen oocytes was extremely significantly higher than that in the fresh group (*p* < 0.01), and the addition of spermidine during the freezing process was able to rescue the proportion of mitochondrial abnormality distribution to a great extent and reduce it to a level that was not significantly different from that in the fresh group (Figure 7A,B). In terms of mitochondrial reactive oxygen species (ROS) levels, there was a tendency for mitochondrial ROS levels to increase in vitrified-frozen oocytes compared with the fresh group (*p* > 0.05), while there was a tendency for them to decrease after the addition of spermidine during the freezing process (*p* > 0.05). On the other hand, mitochondrial ROS levels were not statistically different between the fresh, vitrified, and spermidine-treated groups (V + Spd) (Figure 7C,D). The abnormal distribution of mitochondria with increased mitochondrial ROS levels after vitrification freezing may suggest a possible impairment of mitochondrial function in vitrification-frozen oocytes. So, mitochondrial membrane potential levels were explored next. The mitochondrial membrane potential of vitrified frozen oocytes was significantly reduced compared with the fresh group (*p* < 0.01). However, the addition of spermidine during the freezing process (V + Spd) significantly increased the mitochondrial membrane potential of vitrified frozen oocytes (*p* < 0.01). Importantly, the addition of spermidine restored the level of membrane potential to a point that was non-significantly different from that of the Fresh group (Figure 8A,B). ATP levels were significantly reduced after vitrification freezing compared with the fresh group (*p* < 0.05), and the addition of spermidine restored ATP levels to levels that were not significantly different from those of the control group (Figure 9A,B).

### 3.6. Spermine Ameliorates Abnormal Endoplasmic Reticulum and Spindle Distribution After Vitrification Freezing

**Figure 10:**
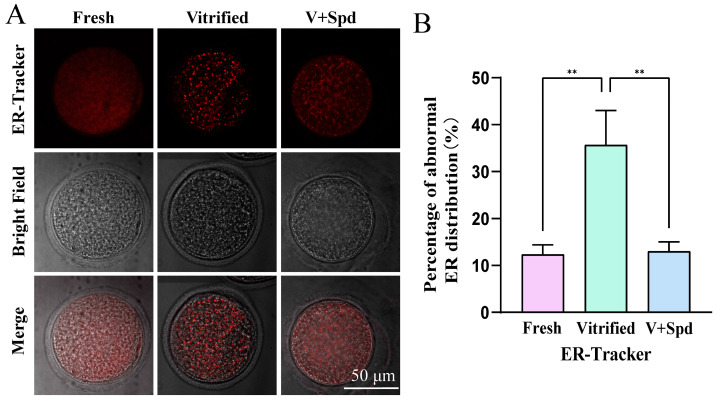
Effect of spermidine on endoplasmic reticulum distribution in vitrified oocytes: (**A**) Even distribution of fluorescence in the cytoplasm of oocytes is considered to be a normal endoplasmic reticulum distribution as shown in fresh group, while clusters of fluorescence or uneven distribution in the cytoplasm are considered to be an abnormal endoplasmic reticulum distribution as shown in vitrified group. (**B**) Histogram of fluorescence intensity statistics for each group, showed in Mean ± SD. ** indicates *p* < 0.01.

**Figure 11:**
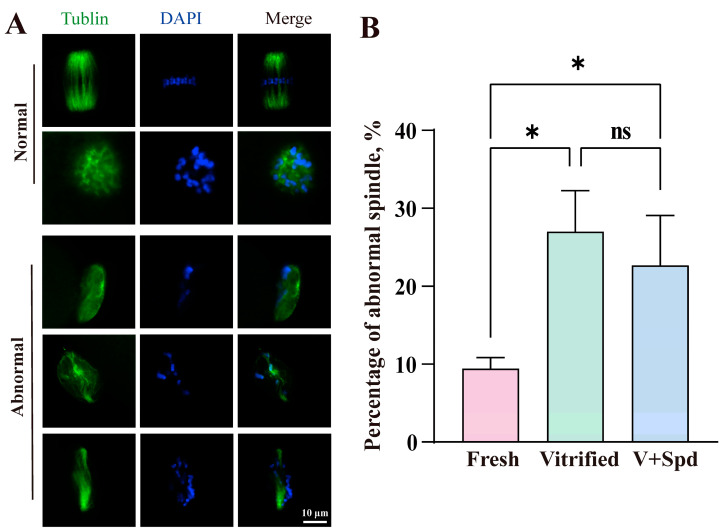
Effect of spermidine on spindle morphology of vitrified oocytes: (**A**) The spindle is visualised using the anti-α-tubulin-FITC antibody, with the figure depicting two normal spindle morphologies, as well as three abnormal spindle morphologies. (**B**) The proportion of abnormal spindle bodies in each group was counted and expressed using Mean ± SD. ns indicates *p* > 0.05, * indicates *p* < 0.05.

Enrichment analyses definitively revealed that vitrification freezing caused severe effects on the pathways of protein ubiquitination, protein dephosphorylation, chromatin organisation, cell division, and regulation of actin cytoskeleton (Figure 4B and Figure 5A–C), while the endoplasmic reticulum and spindle were the main sites and locations of these pathways. We explored the endoplasmic reticulum distribution and spindle morphology. As shown in Figure 10, the proportion of abnormal endoplasmic reticulum distribution in vitrified-frozen oocytes was significantly higher than that in fresh group oocytes. However, spermidine treatment led to a significant reduction in endoplasmic reticulum distribution abnormalities, approaching levels comparable to those observed in the fresh-group oocytes. We also performed immunofluorescence staining of spindle morphology. As Figure 11 shows, the normal spindle was a bipolar structure with chromosomes regularly arranged on the equatorial plate, while the chromosomes of the abnormal spindle were arranged haphazardly and appeared as multipolar or unipolar spindle. The results showed that abnormal spindle formation increased significantly after vitrification freezing (*p* < 0.05), and the abnormal spindle ratio tended to decrease after the addition of spermidine (*p* > 0.05).

### 3.7. Effect of Spermidine on Calcium Homeostasis in Frozen Oocytes

**Figure 12:**
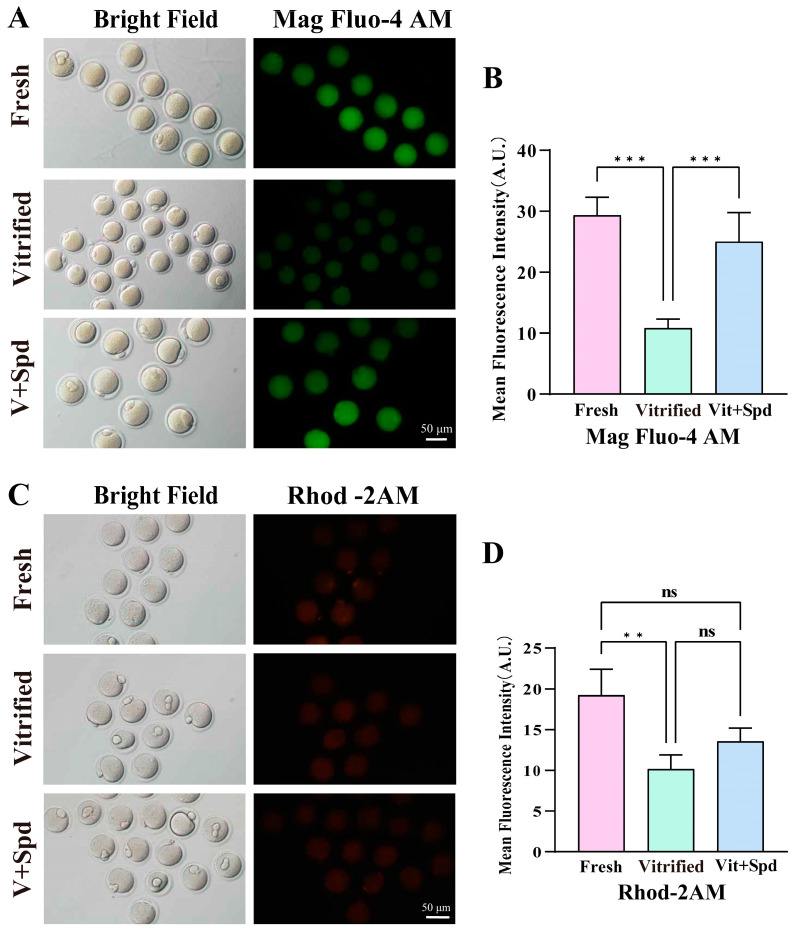
Effect of spermidine on calcium homeostasis in vitrified frozen mouse oocytes: (**A**) Total intra-oocyte calcium levels were visualised using the Mag Fluo-4 AM probe. Higher fluorescence intensity indicates higher intracellular calcium levels. (**C**) Mitochondrial-specific calcium levels in oocytes were visualised using the Rhod-2 AM probe. Stronger fluorescence intensity indicates higher calcium levels within the mitochondria. (**B**,**D**) Histogram of fluorescence intensity statistics for each group, showed in Mean ± SD. ns indicates *p* > 0.05, ** indicates *p* < 0.01. *** indicates *p* < 0.0001.

Calcium homeostasis is vital for normal oocyte development, and intracellular calcium ion homeostasis is a key factor in successful oocyte meiosis and fertilisation [22]. Enrichment analyses revealed significant abnormalities in the Phosphatidylinositol 3-Kinase—Protein Kinase B signalling pathway(PI3K-Akt), autophagy, mitophagy, and regulation of actin cytoskeleton (Figure 4B,D and Figure 5A–C). Meanwhile, endoplasmic reticulum and mitochondria, the two major intracellular calcium reservoirs, were both similarly damaged in vitrification freezing (Figure 7, Figure 8, Figure 9 and Figure 10). We therefore analysed total intra-oocyte calcium levels and mitochondrial-specific calcium levels. The quantitative analysis of total intra-oocyte calcium content demonstrated a significant reduction in vitrified oocytes relative to fresh controls, indicating compromised calcium homeostasis following cryopreservation (Figure 12). However, the total intra-oocyte calcium levels of frozen oocytes were significantly increased after the addition of spermidine during vitrification freezing (Figure 12A) (*p* < 0.01). On the other hand, examination of mitochondrial-specific calcium levels revealed that mitochondrial calcium levels in vitrified frozen oocytes were extremely significantly lower (*p* < 0.01) compared with those in the fresh group. However, there was a tendency for mitochondrial calcium levels in frozen oocytes to increase after the addition of spermidine during the vitrification-freezing process (*p* > 0.05).

### 3.8. Effect of Spermidine on Meiosis in Frozen Oocytes

**Figure 13:**
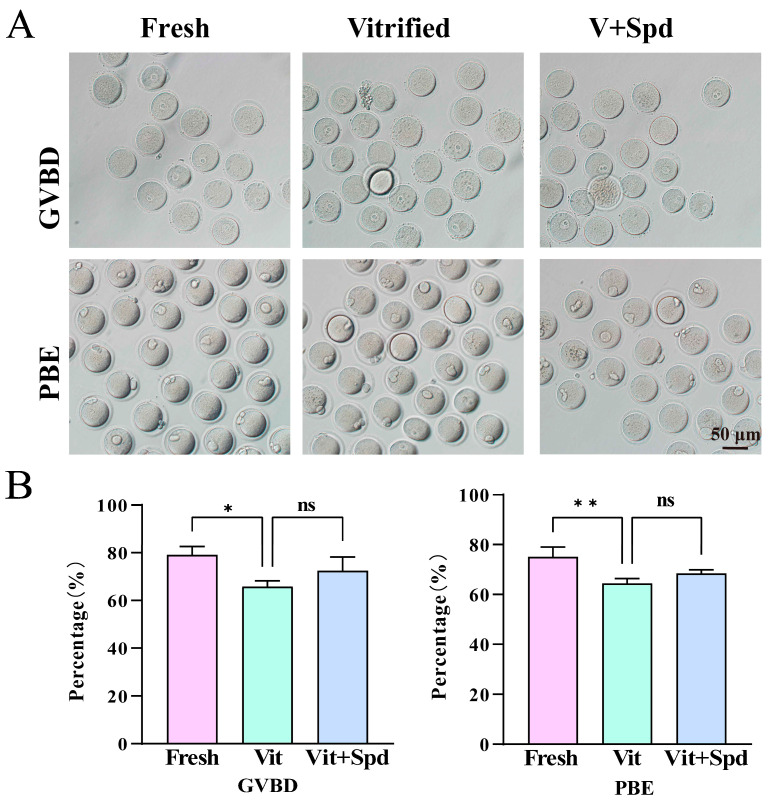
Effect of spermidine on the in vitro maturation of frozen oocytes: (**A**) Microscopic photographs of the germinal vesicle breakdown and the expulsion of the first polar body in the fresh, vitrified, and V + Spd groups. (**B**) Statistical histograms of germinal vesicle breakdown and first polar body expulsion for fresh, vitrified, and V+Spd groups, shown in Mean ± SD. ns indicates *p* > 0.05, * indicates *p* < 0.05, and ** indicates *p* < 0.01.

Our enrichment analysis also revealed abnormalities in cell cycle and cell-division-related pathways. Next, we investigated whether Spd could alleviate the meiotic damage caused by vitrification freezing on mouse oocytes. We performed vitrification freezing of germinal vesicle (GV)-stage oocytes, added 50 μmol/L Spd during in vitro maturation after warming, and then assessed the ratio of germinal vesicle breakdown (GVBD) and polar body extrusion (PBE) of oocytes in each group (Figure 13A,B). The results showed that the proportion of GVBD in frozen oocytes was significantly lower compared with the fresh group (*p* < 0.05). However, the addition of Spd did not significantly improve the occurrence of GVBD (*p* > 0.05). On the other hand, the rate of PBE was extremely significantly reduced after vitrification freezing (*p* < 0.01), but the addition of Spd also had no significant effect on the rate of PBE (*p* > 0.05).

## 4. Discussion

Cryopreservation of oocytes is a significant method of germplasm resource conservation. However, oocytes are characterised by their large size (>70 μm) and volume, which render their cryopreservation particularly challenging [23]. Conversely, it has been demonstrated that freezing leads to a substantial accumulation of intracellular reactive oxygen species, which subsequently induces significant oxidative stress within the oocytes and results in the destruction of their developmental capacity [24]. Furthermore, the process of freezing has been demonstrated to cause the collapse of the cytoskeleton and the disruption of the cellular barrier, thereby accelerating the process of cell death [25]. Consequently, the development of a straightforward yet efficacious technique to mitigate the deleterious effects of vitrification freezing is imperative.

Spermidine, a class of polyamines found in eukaryotes, has been demonstrated to regulate autophagy, apoptosis, oxidative stress, and other processes [26]. It is important to note that spermidine plays an indispensable role in cellular function. Spermidine has been shown to enhance mitochondrial energy supply by activating mitochondrial trifunctional protein (MTP) in CD8+ T cells of aged mice [27]. Furthermore, exogenous supplementation of spermidine has been demonstrated to significantly enhance the antioxidant capacity of murine ovarian and follicular granulosa cells, reduce ovarian lipid peroxidation, and decrease follicular atresia [28]. Moreover, spermidine has been demonstrated to regulate BCL-1 and LC3-II proteins, thereby mitigating the oxidative stress induced by hydrogen peroxide and inducing mitochondrial autophagy, which in turn prevents oxidative DNA damage caused by oxidative stress [29]. Despite the significance of spermidine, the role of spermidine in cryoprotection remains to be extensively studied.

The present study was initiated with the objective of investigating the effects of spermidine on the survival of oocytes and the development of in vitro fertilisation. The results demonstrated that the incorporation of 50 μmol/L spermidine during the vitrification process led to a substantial enhancement in oocyte survival and in vitro development. Subsequently, a comprehensive transcriptome analysis was conducted on MII oocytes from the frozen, spermidine-added, and control groups to elucidate the underlying mechanisms by which spermidine addition affects vitrified oocytes. The results indicated substantial variation among the oocytes in their principal components. A substantial number of pathways and genes were found to be aberrant; however, 47.3% of the aberrant pathways and 43.3% of the aberrantly expressed genes were rescued following the addition of spermidine.

We then proceeded to conduct further research into the effect of spermidine on oxidative stress in frozen oocytes. Under normal conditions, cells are able to regulate the oxidative stress occurring intracellularly through their own antioxidant mechanisms [30], and intracellular glutathione acts as a scavenger for scavenging oxygen-free radicals in the cell, thereby protecting the cell from oxidative stress damage [31]. The results of the present experiment demonstrated a highly significant decrease in intracellular glutathione levels after freezing, along with a significant increase in intracellular reactive oxygen species levels. These results are similar to those of a previous study in porcine oocytes during freezing [32]. This finding serves to reinforce the notion that the alterations in oxidative stress levels triggered by freezing are a shared characteristic amongst diverse mammalian species. Mitochondria, as the most significant site of energy production in the cell, are imperative for oocyte maturation, fertilisation, and early embryonic normal development [33]. It has been demonstrated that goat GV-stage oocytes were frozen with abnormal mitochondrial content and intra-mitochondrial reactive oxygen radical levels, which ultimately led to the failure of the oocytes to develop normally [34]. These results are similar to those of the present study. This finding underscores the significance of safeguarding the normal function of mitochondria in frozen oocytes, as a pivotal strategy to ensure the subsequent development of oocytes in all mammals. Abnormal mitochondrial membrane potential is an indication of early apoptosis, and a significant body of research has confirmed a substantial reduction in mitochondrial membrane potential in oocytes following vitrification freezing [35]. Furthermore, Zhang [19] found that spermidine could restore or increase mitochondrial membrane potential levels that decrease due to ageing. The present study found that the mitochondrial membrane potential level of the spermidine-added group was considerably higher than that of the frozen control group. This suggests that the role of spermidine in improving mitochondrial membrane potential may be independent of the biological samples and is instead a property of spermidine itself. However, further experimentation is required to verify this. During the freezing process of porcine oocytes, it has been demonstrated that a significant amount of reactive oxygen species is released due to the disruption of mitochondrial function, leading to oxidative stress in porcine oocytes [36]. However, in this study, a tendency towards an increase in the level of reactive oxygen species in the mitochondria of mouse oocytes after freezing was observed, though this increase did not reach statistical significance. This discrepancy may be attributed to the reduced lipid content of mouse oocytes compared to porcine oocytes, which results in a diminished production of ice crystals during the freezing process, thereby minimising the mechanical damage to the mitochondria. The endoplasmic reticulum is a vital component of oocytes, responsible for regulating calcium ion homeostasis and performing essential physiological functions, including protein synthesis and modification [37]. In this study, we observed that the distribution of the endoplasmic reticulum was abnormal in oocytes following freezing. However, the proportion of this abnormal distribution was reduced after the addition of spermidine. A previous study demonstrated that exogenous spermidine addition could inhibit endoplasmic reticulum stress and the Wnt-signalling pathway, thereby attenuating myocardial fibrosis [38]. Consequently, the present study hypothesises that the activation of the endoplasmic reticulum stress response, induced by freezing, may be a causative factor in the disruption of the endoplasmic reticulum structure during the vitrification of mouse oocytes. The addition of spermine has been shown to inhibit endoplasmic reticulum stress, promote autophagy, and regulate protein synthesis and modification processes (Figure 4 and Figure 5). However, the specific mechanism remains to be elucidated. Calcium homeostasis plays an important role in oocyte quality and embryonic developmental competence [39]. Previous studies have found that the cryoprotectant DMSO acts on oocytes and organelle membranes, resulting in the release of calcium ions stored in the endoplasmic reticulum or mitochondria into the cytosol, and an increase in the intracellular concentration of calcium ions, which results in the hardening of the zona pellucida, and ultimately leads to a decrease in the fertilisation rate of the oocyte and the developmental competence of the embryo [22]. And this study found that the use of Spd as a supplement can improve this abnormality (Figure 12). Finally, this study also found that the proportion of abnormal spindles increased significantly after freezing, but the addition of spermidine did not improve this phenomenon. It can be hypothesised that, given that the spindle is initially formed at the MII stage, the spindle of MII-stage oocytes is more sensitive to low temperatures [40], and thus, the severe damage to actin by freezing could not be rescued by spermidine alone. The precise underlying mechanisms, however, remain to be elucidated. In general, our results identified a supplement that not only improves the level of oxidative stress but also effectively improves mitochondrial and endoplasmic reticulum distribution during vitrification-freezing process of mouse oocytes. However, whether this excellent effect can do the same in other mammals needs to be tried and validated on a large scale in, e.g., sheep, cattle, or even human oocyte vitrification-freezing processes.

## 5. Conclusions

The present study demonstrates that vitrification freezing induced significant deleterious effects on oocyte survival and subsequent embryonic developmental capacity. However, the addition of spermidine resulted in a substantial improvement in the survival of post-freezing oocytes and enhancement of their embryonic developmental capacity. Further transcriptome analyses revealed that spermidine rescued, to a certain extent, multiple biological pathways affected by freezing, including those associated with oxidative stress, mitochondrial function, endoplasmic reticulum function, and calcium homeostasis. Moreover, immunofluorescence experiments provided evidence that spermidine mitigates the damage caused by freezing to oocytes by improving these critical cellular functions. Consequently, the findings of this study not only provide a new framework for the optimisation of vitrification freezing techniques but also offer potential intervention strategies to improve the survival and embryonic developmental capacity of oocytes after freezing.

## Figures and Tables

**Table 1:** Effect of spermidine on the survival of oocytes after vitrification.

Groups (μmol/L)	Total Number of Oocytes	Number of Surviving Oocytes	Survival Rate (%)
0	357	293	82 ± 3 ^Bb^
25	309	260	83 ± 9 ^Bb^
50	364	331	92 ± 4 ^Aa^
100	335	220	66 ± 4.2 ^Cc^

The same letter on the shoulder indicates non-significant difference (*p* > 0.05), different lowercase letters indicate significant difference (*p* < 0.05), and different capital letters indicate highly significant difference (*p* < 0.01).

**Table 2:** Effect of spermidine on embryonic development after in vitro fertilisation of vitrified oocytes.

Groups	The Number of Prokaryotic Embryos	Cleavage Rate (%)	Blastocyst Rate (%)
Fresh Control	196	86.22 ± 2 ^Aa^	47.33 ± 5 ^Aa^
Vitrified	234	68.8 ± 4 ^Bb^	6 ± 3 ^Bc^
Spd 50 μmol/L	368	68.09 ± 10 ^Bb^	14.86 ± 7 ^Bb^

The same letter on the shoulder indicates non-significant difference (*p* > 0.05), different lowercase letters indicate significant difference (*p* < 0.05), and different capital letters indicate highly significant difference (*p* < 0.01).

## Data Availability

The data presented in this study are available on request from the corresponding author.
